# Corosolic acid ameliorates cardiac hypertrophy via regulating autophagy

**DOI:** 10.1042/BSR20191860

**Published:** 2019-12-04

**Authors:** Zhao-Peng Wang, Difei Shen, Yan Che, Ya-Ge Jin, Sha-Sha Wang, Qing-Qing Wu, Heng Zhou, Yan-Yan Meng, Yuan Yuan

**Affiliations:** 1 Department of Cardiology, Renmin Hospital of Wuhan University, Wuhan, China; 2 Hubei Key Laboratory of Metabolic and Chronic Diseases, Wuhan, China

**Keywords:** AMPK, autophagy, cardiac hypertrophy, corosolic acid

## Abstract

**Aim:** In this work, we explored the role of corosolic acid (CRA) during pressure overload-induced cardiac hypertrophy. **Methods and results:** Cardiac hypertrophy was induced in mice by aortic banding. Four weeks post-surgery, CRA-treated mice developed blunted cardiac hypertrophy, fibrosis, and dysfunction, and showed increased LC3 II and p-AMPK expression. In line with the *in vivo* studies, CRA also inhibited the hypertrophic response induced by PE stimulation accompanying with increased LC3 II and p-AMPK expression. It was also found that CRA blunted cardiomyocyte hypertrophy and promoted autophagy in Angiotensin II (Ang II)-treated H9c2 cells. Moreover, to further verify whether CRA inhibits cardiac hypertrophy by the activation of autophagy, blockade of autophagy was achieved by CQ (an inhibitor of the fusion between autophagosomes and lysosomes) or 3-MA (an inhibitor of autophagosome formation). It was found that autophagy inhibition counteracts the protective effect of CRA on cardiac hypertrophy. Interestingly, AMPK knockdown with AMPKα2 siRNA-counteracted LC3 II expression increase and the hypertrophic response inhibition caused by CRA in PE-treated H9c2 cells. **Conclusion:** These results suggest that CRA may protect against cardiac hypertrophy through regulating AMPK-dependent autophagy.

## Introduction

Cardiac hypertrophy is an initially adaptive response after pressure or volume overload, but prolonged cardiac hypertrophy contributes to the development of heart failure. Numerous intracellular signaling pathways and processes have been characterized as important intervention target as preventing or treating cardiac hypertrophic and heart failure. Autophagy, an intracellular self-degradative mechanism, plays an essential role in degrading and recycling cytosolic proteins and organelles [1]. There is a close relation between cardiomyocyte autophagy and cardiac hypertrophy [2]. In the moderate transverse aortic constriction model, both up-regulation of autophagy and down-regulation of protein synthesis are involved in regression of cardiac hypertrophy [3]. Now it is generally considered that cardiac autophagosome–lysosome pathway (ALP) insufficiency occurs in sustained cardiac hypertrophy as seen in chronic pressure overload-induced left ventricular hypertrophy, especially during the progression from adaptive cardiac hypertrophy to heart failure. The ALP activation is beneficial to the heart. The ALP insufficiency caused by either inadequate autophagosome formation or impaired autophagosome clearance during the pressure overload progression that may contribute to the maladaptation of pressure overload-induced cardiac hypertrophy and the development of heart failure [4].

Corosolic acid (CRA) is a pentacyclic triterpene existing naturally in various medicinal plants including Vaccinium macrocarpon, Ugni molinae, Eriobotrya japonica, and etc. [5]. Human studies noted that CRA decreases fasting as well as postprandial blood glucose levels in humans [5]. Various animal studies showed that CRA exhibits numerous pharmacological properties, such as anti-angiogenic [6], anti-diabetic [7], antioxidative, anti-proliferative, anti-tumoral [8], and anti-obesity [9] activities. A recent study demonstrated that CRA may protect the liver against ethanol-induced injury by modulation of MAPK signaling and autophagy activation, connecting CRA with autophagy [10]. The effects of CRA on cardiac autophagy under pressure overload are currently unknown.

The objective of the present study is to examine the effect of CRA on cardiac hypertrophy autophagy process. We used an *in vivo* pressure overload-induced cardiac hypertrophy model and an *in vitro* norepinephrine (PE)-induced cardiomyocyte hypertrophy model to address this issue. We postulate that CRA could retard pressure overload-induced cardiac hypertrophy and its effects correlates with cardiac autophagy.

## Materials and methods

### Reagent

CRA was purchased from Jianfeng Natural Product R&D Co., Ltd (Tianjin, China). The autophagy inhibitor Chloroquine diphosphate (CQ) was obtained from ABCAM, U.S.A., and was applied to cardiomyocytes at a concentration of 10 μM. The autophagy inhibitor 3-methyladenine (3-MA) was obtained from Selleck, U.S.A., and was applied to cardiomyocytes at a concentration of 10 mM. AMPK siRNA was purchased from Guangzhou RiboBio Co., LTD (Guangzhou, China).

### Animals

The present study conforms with the guidelines of the Animal Care and Use Committee of Renmin Hospital of Wuhan University and was performed in accordance with the Guide for the Care of Laboratory Animals published by the US National Institutes of Health (NIH Publication No.85-23, revised 1996). All animal experimental procedures in the study were approved by the Animal Care and Use Committee of Renmin Hospital of Wuhan University (approval number: 20170510). All animal experimental procedures were conducted in Cardiovascular Research Institute of Wuhan University (Wuhan, China). Mice were initially anaesthetized with 1.5% isoflurane using a rodent ventilator. Cardiac hypertrophy was generated in male C57 *BL/6J* mice (8–10 weeks of age) by aortic banding (AB) as described previously [11]. Sham-treated mice underwent the same surgical procedures except the descending aorta was not constricted. Mice were assigned to four groups: a saline-treated group with Sham surgery (Sham, *n* = 14), a saline-treated group with AB surgery (AB+CRA 0, *n* = 14), a CRA (10 mg/kg/d)-treated group with AB surgery (AB+CRA 10, *n* = 14), and a CRA (20 mg/kg/d)-treated group with AB surgery (AB+CRA 20, *n* = 14). Treatments were started 2 weeks before surgery, and were administered by daily irrigation for 6 weeks. Four weeks post-surgery, all mice underwent echocardiography and catheter-based measurements of hemodynamic parameters. After the invasive hemodynamic measurements, mice were killed by cervical dislocation.

### Echocardiography

Echocardiography was carried out as previously described [11]. Briefly, the left ventricle (LV) dimensions were assessed in parasternal short-axis view during systole or diastole. LV end-systolic diameter (LVESD), ejection fraction (EF), and fraction shortening (FS) were obtained from the LV M-mode tracing with a sweep speed of 50 mm/s at the mid-papillary muscle level.

### Catheter-based measurements of hemodynamic parameters

Cardiac catheterization was carried out as previously described [11]. Briefly, a microtip catheter transducer (SPR-839, Millar Instruments, Houston, TX, U.S.A.) was inserted into the left ventricle via the right carotid artery after anaesthetization. The signals were recorded using a Millar Pressure-Volume System (MPVS-400, Millar Instruments, Houston, TX, U.S.A.), and the endsystolic volume (ESV), end-diastolic volume (EDV), maximal rate of pressure development (d*P*/d*t* max), maximal rate of pressure decay (d*P*/d*t* min), end-systolic pressure (ESP), and end-diastolic pressure (EDP) were analyzed using the PVAN data analysis software.

### Histological analysis

Histological analysis was carried out as previously [11]. Tissue sections from each group were cut at 4–5 μm and mounted onto slides and were conducted to Hematoxylin and eosin (H&E) and Picrosirius Red (PSR) staining. Tissue sections were visualized by light microscopy. A single myocyte was measured with a quantitative digital image analysis system (Image Pro-Plus, version 6.0).

### Quantitative real-time RT-PCR

The relative mRNA expression of atrial natriuretic peptide (ANP), B-type natriuretic peptide (BNP), β-myosin heavy polypeptide (β-MHC), α-myosin heavy polypeptide (α-MHC), fibronectin, connective tissue growth factor (CTGF), Collagen Iα, and interleukin-6 (IL-6) were examined using Quantitative Real-time RT-PCR. As reported [11], RNA was collected from left ventricular tissue using TRIzol (Invitrogen, 15596-026), and reverse transcribed into cDNA for real-time PCR analysis using oligo (DT) primers and the Transcriptor First Strand cDNA Synthesis Kit (Roche, 04896866001). cDNA was synthesized from 2 μg of total RNA. The PCR amplifications were quantified using a LightCycler 480 SYBR Green 1 Master Mix (Roche, 04707516001) and the results were normalized against glyceraldehyde-3-phosphate dehydrogenase (GAPDH) gene expression.

### Western blotting

Myocardium or cardiomyocytes were lysed in RIPA lysis buffer, and the protein concentration was measured with the BCA protein assay kit (Themo, 23227) by an ELISA reader (Synergy HT, Bio-tek). Protein lysates were electrophoresed on 10% SDS-PAGE gels, transferred onto polyvinylidene difluoride (PVDF) membranes (Millipore, IPFL00010), and blocked with 5% non-fat milk for 2 h. The PDVF membranes were incubated with the appropriate primary antibodies. On the second day, the membranes were washed three times with phosphate-buffered saline (PBS) and incubated with secondary antibodies for 1 h. The membranes were then scanned and analyzed with a two-colour infrared imaging system (Odyssey, U.S.A.). Protein expression levels of AMP-activated protein kinase (AMPK), phosphorylated AMPK (P-AMPK), 4EBP1, P-4EBP1, LC3, and β-MHC (Cell Signaling Technology) were normalized to the GAPDH (Santa Cruz).

### Cardiomyocyte cultures

Cardiomyocyte culture was carried out as previously reported [12]. In detail, the H9c2 cells (Cell Bank of the Chinese Academy of Sciences, Shanghai, China) were seeded in the high-glucose Dulbecco’s modified Eagle’s medium (GIBCO, C11995) containing 10% fetal bovine serum (GIBCO, 10099) in humidified CO_2_ incubator (SANYO 18M) with 5% CO_2_ at 37°C. Cells at exponential growth were dissociated with 0.25% trypsin (GIBCO, 25200) and were seeded in six-well culture plates at a seeding density of 1 × 10^6^/well before being incubated for 24 h. Then cells were cultured with serum-free DMEM for another 12 h. CRA was prepared in sterilized dimethyl-sulfoxide (DMSO, Sigma) and stored at 4°C. PE (Sigma) (1 μM) in the presence or absence of different concentrations of CRA (1, 5, and 10 μM), and the cells were incubated for 24 h. And the cells were collected for the following analyses. To evaluate the effect of autophagy on the CRA-treated cardiomyocyte hypertrophy, we applied the autophagy inhibitor CQ (10 μM) or 3-MA (10 mM) to the experiments. Cells were assigned to eight groups first: CON, CON+CRA, PE, PE+CRA, CON+CQ, CON+CRA+CQ, PE+CQ, and PE+CRA+CQ, and the cells were collected for the following analyses. Then the cells were assigned to the next eight groups: CON, CON+CRA, PE, PE+CRA, CON+3-MA, CON+CRA+3-MA, PE+3-MA, and PE+CRA+3-MA.

The cells were incubated with Ang II (Sigma) (1 μM) in the presence or absence of CRA (10 μM) for 24 h. And the cells were collected for the following analyses. To evaluate the effect of autophagy on the CRA-treated cardiomyocyte hypertrophy, we applied the autophagy inhibitor 3-MA (10 mM) to the experiments. The cells were assigned to the next eight groups: CON, CON+CRA, Ang II, Ang II +CRA, CON+3-MA, CON+CRA+3-MA, Ang II +3-MA, and Ang II +CRA+3-MA.

### α-Actinin staining

To identify the cardiomyocytes and assess cardiomyocyte hypertrophy, immunocytochemistry for cardiac α-actinin was performed according to our previous study [11]. Briefly, the cells were washed with PBS, fixed with RCL2 (ALPHELYS, RCL2-CS24L), permeabilized in 0.1% Triton X-100 in PBS, and stained with anti-α-actinin (Millipore, 05-384) at a dilution of 1:100 in 1% goat serum. The secondary antibody was Alexa Fluor® 568 goat anti-mouse IgG (Invitrogen, A11004). The cardiomyocytes on coverslips were mounted onto glass slides with SlowFade Gold antifade reagent with DAPI (Invitrogen, S36939).

### Evaluation of tandem fluorescent LC3 puncta

H9c2 cardiomyocytes were transfected with adenovirus harbouring mCherry-GFP-LC3 at an MOI of 50 for 8 h. Then, the cardiomyocytes were exposed to PE stimuli in the presence or absence of different concentrations of CRA (1, 5, and 10 μM) for an additional 24 h. After fixation with 4% paraformaldehyde, DAPI was used to stain nuclei, images were captured using fluorescence microscope.

### Cardiomyocyte transfections

The knockdown of AMPKα1 or AMPKα2 was achieved by small interfering RNA (siRNA) transfection performed with riboFECT™ CP Transfecton Kit according to the manufacturer’s instructions. The sequence of siRNA targeting AMPKα1 was the following: GCATATGCTGCAGGTAGAT. The sequence of siRNA targeting AMPKα2 was the following: GCTGTGGATCGCCAAATTA. Twenty-four hours after seeding, the H9c2 cells were transfected with AMPKα1-specific siRNA (100 nmol/l), AMPKα2-specific siRNA (100 nmol/l), or scramble siRNA (100 nmol/l) for 12 h. After an additional 12 h of serum deprivation after transfection, the cardiomyocytes were exposed to PE stimuli at 1 μM for 24 h.

### Statistical analysis

Data are presented as mean ± SEM. Statistical analysis was performed using SPSS 13.0 (SPSS Inc.) software. Data were analyzed by one-way ANOVA followed by Tukey’s post-hoc test. Values of *P* < 0.05 were considered significant.

## Results

### CRA ameliorates pressure overload-induced cardiac hypertrophy and left ventricular dysfunction

**Figure 1:**
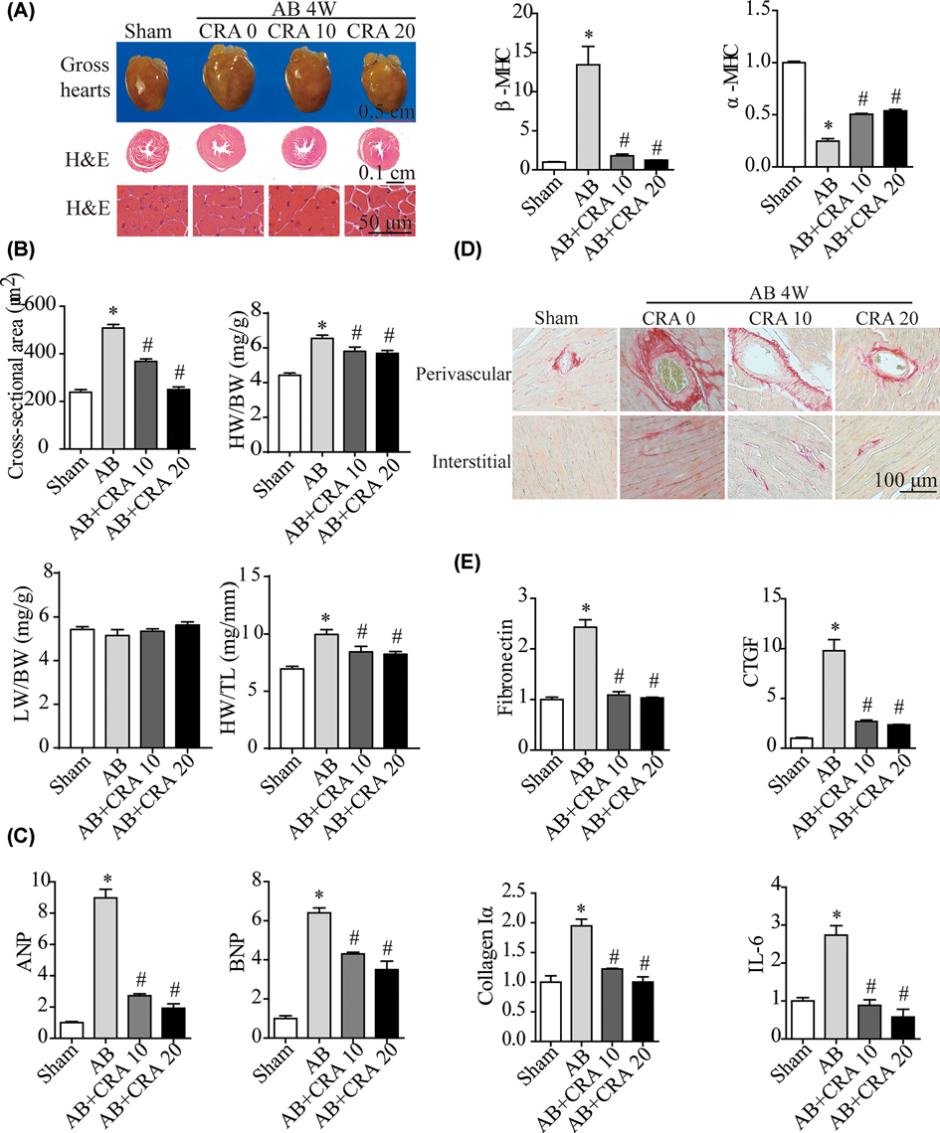
CRA ameliorates pressure overload-induced cardiac hypertrophy (**A**) Representative gross heart, HE-stained heart sections of mice in the indicated groups (*n* = 5–8 mice per experimental group). (**B**) Quantification of CSA in each group (*n* = 100–200 cells per experimental group), and HW/BW, HW/TL, LW/BW ratios of mice in each group (*n* = 11–17 mice per experimental group). (**C**) Levels of cardiac hypertrophy-related transcripts (ANP, BNP, β-MHC, α-MHC) quantified by RT-PCR (*n* = 6 mice per experimental group). (**D**) PSR staining for detecting fibrosis (*n* = 5–7 mice per group). (**E**) mRNA expression levels of CTGF, Collagen Iα, IL-6 and Fibronectin in each group (*n* = 6 per group). Data are presented as mean ± SEM. *P < 0.05 versus Sham group. #*P* < 0.05 versus AB group.

**Figure 2:**
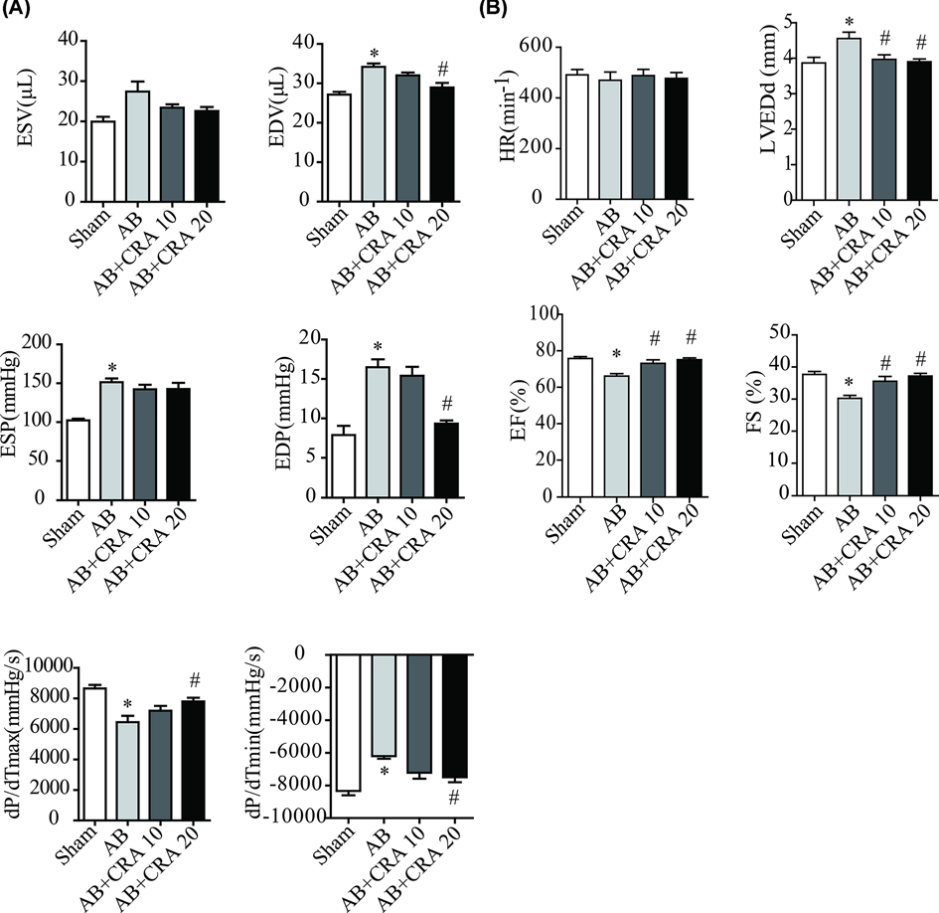
Hemodynamic and echocardiographic parameters of mice in each group (**A**) Hemodynamic parameters (*n* = 7–8 mice per experimental group). (**B**) Echocardiographic parameters. *P < 0.05 versus Sham group. #*P* < 0.05 versus AB group.

Over the period of pressure overload, the mouse heart showed depressed left ventricular contractility and myocardial structural changes, including cellular hypertrophy and interstitial fibrosis. Four weeks of AB treatment induced a hypertrophic phenotype in WT hearts, with significant increases in heart weight/body weight (HW/BW), heart weight/tibial length (HW/TL), cross-sectional area of cardiomyocytes, and left ventricular mRNA levels of hypertrophic markers. In addition, compared with controls, the AB group displayed a higher degree of interstitial fibrosis, increased mRNA expression of fibrotic markers (Figure 1A–E), end diastolic volume (EDV), and end-diastolic diameter (LVEDd) (Figure 2A,B). Changes for these indicators of cardiac hypertrophy, fibrosis and injury were significantly ameliorated for CRA-treated hypertrophic mice (Figure 1A–E).

Cardiac remodeling are also characterized by a decrease in cardiac function, 4 weeks after AB, the degree of TAC was similar among the groups as indicated by end-systolic pressure (ESP) (Figure 2A). As compared with the AB group, CRA-treated mice showed better cardiac contractility as indicated by increased maximal rate of pressure development (d*P*/d*T*_max_) (Figure 2A), ejection fraction (EF), and fractional shortening (FS) (Figure 2B). CRA-treated mice also showed improved cardiac diastolic function as indicated by reduced minimum rate of pressure development (d*P*/d*T*_min_) (Figure 2A).

### CRA protects against the PE and Ang II-induced cardiomyocyte hypertrophy

**Figure 3:**
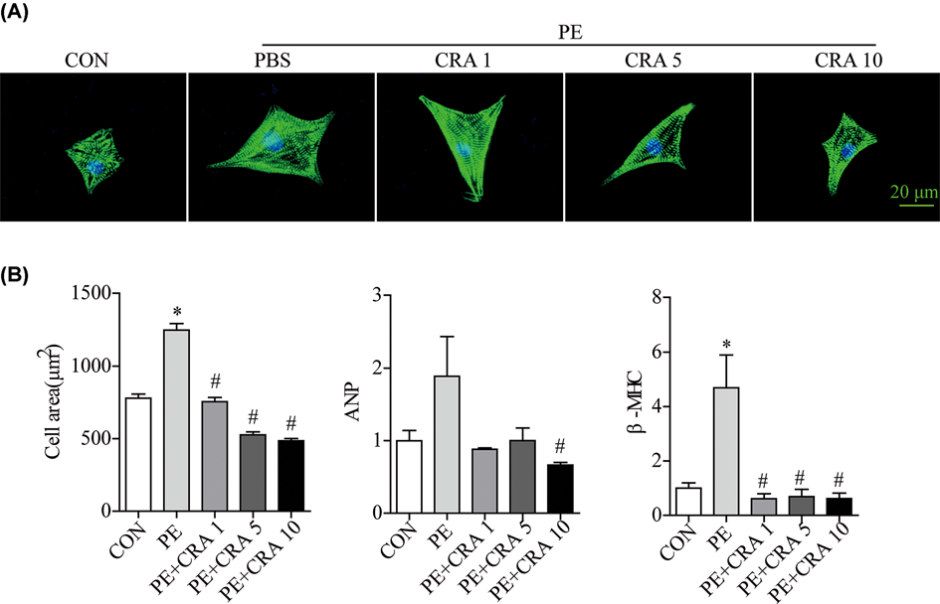
CRA blunts cardiomyocyte hypertrophy in PE-treated H9c2 cells (**A**) Representative imaging of immunostaining H9c2 cells for α-actinin (green) in each group. (**B**) Quantification of cell surface area (*n* = 50^+^ cells per group), ANP, and β-MHC mRNA levels in H9c2 cells in each group. *P < 0.05 versus CON group. #*P* < 0.05 versus PE group.

To investigate the effect of CRA on cardiomyocyte hypertrophy, several indicators were analyzed, including ANP, β-MHC, and cardiomyocyte surface area after 24 h of incubation with PE. As shown in Figure 3A,B, PE enhanced the mRNA expression levels of ANP and β-MHC in cardiomyocytes as well as the cardiomyocyte surface area. CRA treatment significantly decreased the mRNA expression levels of ANP and β-MHC. Similarly, the cardiomyocyte surface area with CRA treatment was markedly smaller as compared with the PE group. What’s more, Ang II enhanced the mRNA expression levels of ANP and β-MHC in cardiomyocytes as well as the cardiomyocyte surface area. CRA treatment promoted autophagy in Ang II-stimulated cardiomyoctes, and also significantly decreased the mRNA expression levels of ANP and β-MHC. Similarly, the cardiomyocyte surface area with CRA treatment was markedly smaller as compared to the Ang II-stimulated group (Supplementary Figure S1A,B).

### CRA activates cardiac autophagy in the process of cardiac hypertrophy

**Figure 4:**
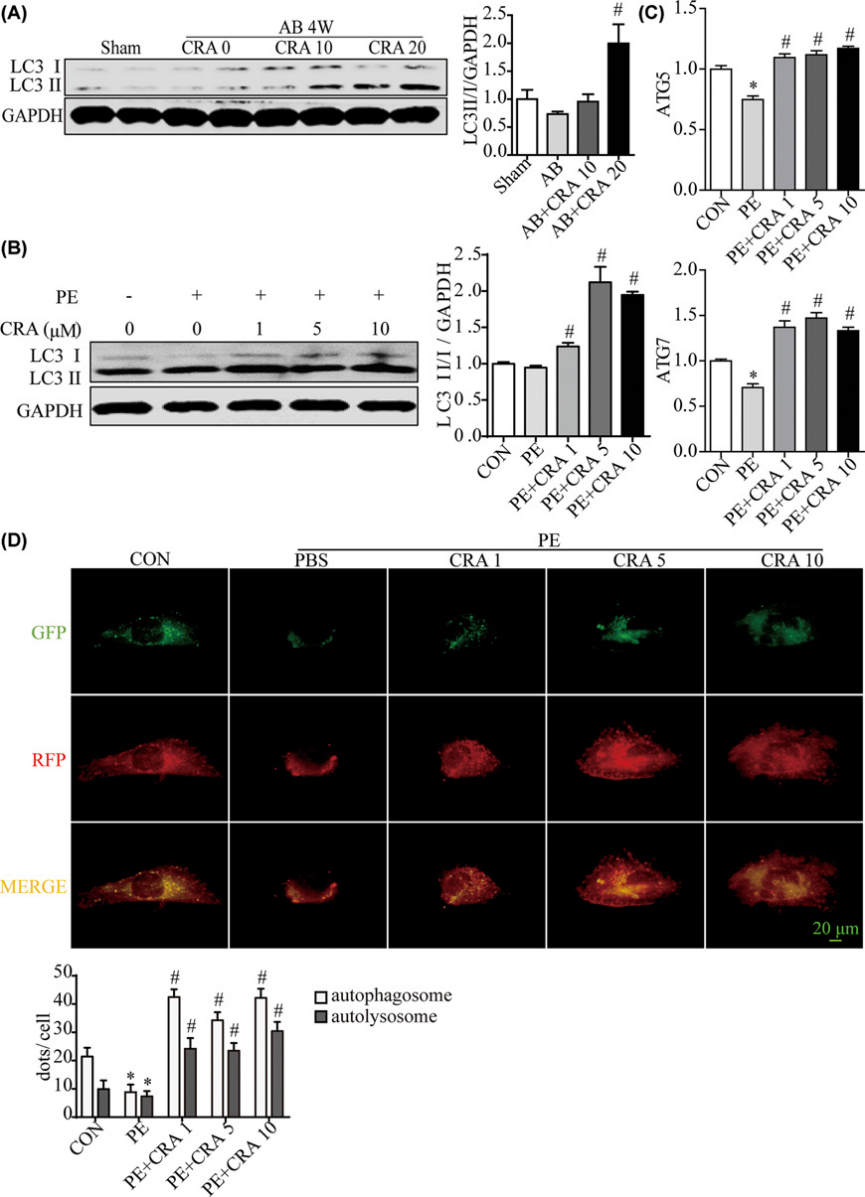
CRA induced autophagy activation in cardiac hypertrophy (**A**) Representative blots and quantitative results for LC3 protein expression in myocardium in each group (*n* = 6 per group). *P < 0.05 versus Sham group. #*P* < 0.05 versus AB group. (**B**) Representative blots and quantitative results for LC3 protein expression in H9c2 cells in each group (*n* = 4 per group). (**C**) ATG5 and ATG7 mRNA expression in H9c2 cells in each group. (**D**) Autophagy was evaluated by transfection with an adenovirus carrying mCherry-GFP-LC3 in each group. *P < 0.05 versus CON group. #*P* < 0.05 versus PE group.

**Figure 5:**
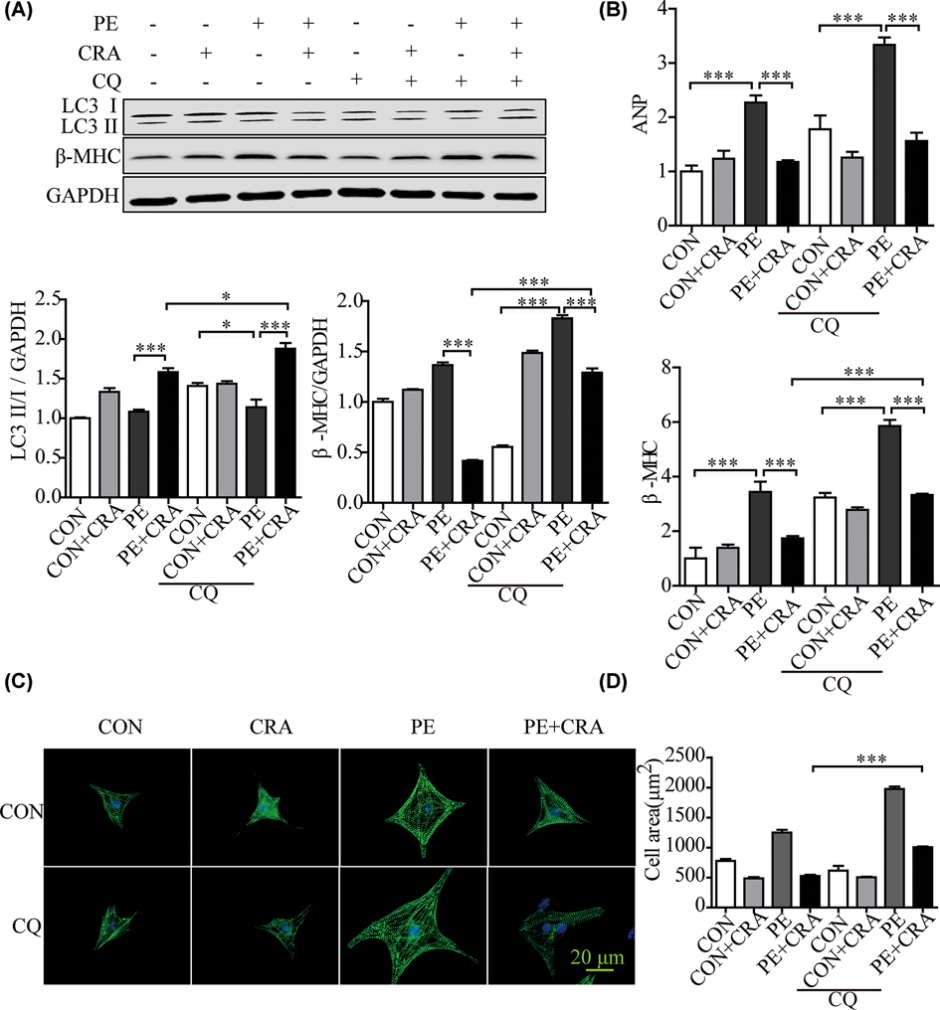
Autophagy inhibition by CQ counteracts the protective effect of CRA on cardiomyocyte hypertrophy (**A**) Representative blots and quantitative results for LC3 and β-MHC protein expression in H9c2 cells in each group (*n* = 4 per group). (**B**) ANP and β-MHC mRNA expression in H9c2 cells in each group. (**C**) Representative imaging of immunostaining H9c2 cells for α-actinin (green) in each group. (**D**) Quantification of cell surface area (*n* = 50^+^ cells per group); *P < 0.05, ***P < 0.0005.

**Figure 6:**
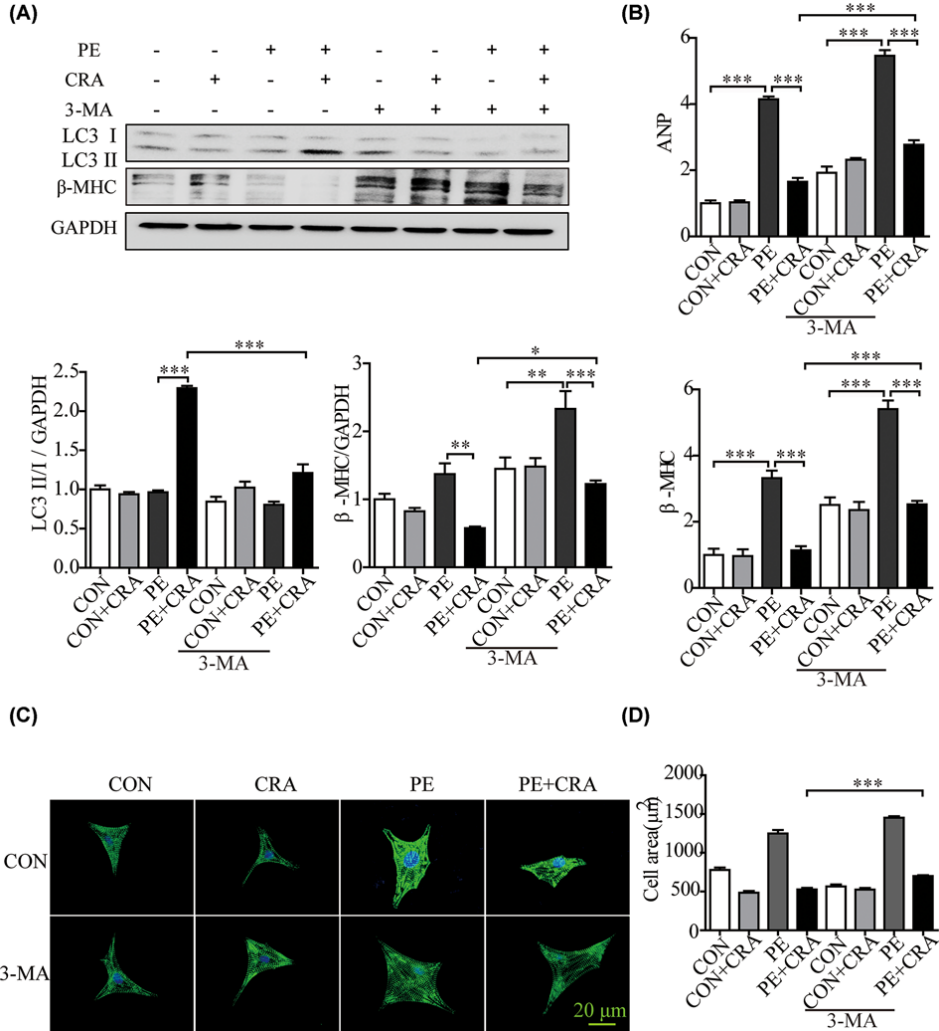
Autophagy inhibition by 3-MA counteracts the protective effect of CRA on cardiomyocyte hypertrophy (**A**) Representative blots and quantitative results for LC3 and β-MHC protein expression in H9c2 cells in each group (*n* = 4 per group). (**B**) ANP and β-MHC mRNA expression in H9c2 cells in each group. (**C**) Representative imaging of immunostaining H9c2 cells for α-actinin (green) in each group. (**D**) Quantification of cell surface area (*n* = 50^+^ cells per group); *P < 0.05, **P < 0.005, ***P < 0.0005.

The amount of LC3 II usually correlates well with the abundance of autophagosomes [4]. We found that LC3 II/I expression to be strongly up-regulated in CRA-treated mice heart after pressure overload (Figure 4A). Consistently, CRA treatment increased LC3 II/I protein expression, ATG5 and ATG7 mRNA expression significantly in PE-treated H9c2 cells, as compared with the PE group (Figure 4B,C). Moreover, autophagy was evaluated by transfection with an adenovirus carrying mCherry-GFP-LC3. The green fluorescent dots indicate autophagosomes while the red fluorescent dots indicate both autophagosomes and autolysosomes. And the yellow dots in the merged images represent autophagosomes [13]. Consistent with the study of the other researchers [13], PE significantly reduced the number of green and yellow fluorescent dots per cell, indicating impaired autophagy in PE-induced cardiomyocyte hypertrophy. In the presence of PE, CRA resulted in increased LC3 II, suggesting of reparative autophagy. Moreover, the number of LC3 dots treated with CRA under PE stimulation increased as compared with the PE group (Figure 4D). It was also found that Ang II increased autophagy in cardiomyocytes, as seen in the Supplementary Figure S1C,D, CRA treatment exaggerated the LC3II/I expression and improved autophagy flux in cardiomyocytes after Ang II stimulation. These results indicated that CRA was helpful for maintaining autophagy in the process of cardiomyocyte hypertrophy. To further verify whether CRA inhibits cardiac hypertrophy by the activation of autophagy, blockade of autophagy was achieved by CQ (an inhibitor of the fusion between autophagosomes and lysosomes) or 3-MA (an inhibitor of autophagosome formation). We assessed the levels of LC3 II in the presence of CQ or 3-MA, and found that as compared with PE+CRA group, PE+CRA+CQ group showed enhanced LC3 II expression; however, PE+CRA+3-MA group displayed decreased LC3 II expression. Interestingly, H9c2 cells in PE+CRA+CQ group and PE+CRA+3-MA group had an exaggerated hypertrophic response based on analyses of hypertrophic markers and cell surface area (Figures 5 and 6), which are hallmarks of cardiomyocyte hypertrophy. These results indicate that autophagy inhibition counteracts the protective effect of CRA on cardiac hypertrophy.

### CRA regulates AMPK pathway in the process of cardiac hypertrophy *in vivo* and *in vitro*

**Figure 7:**
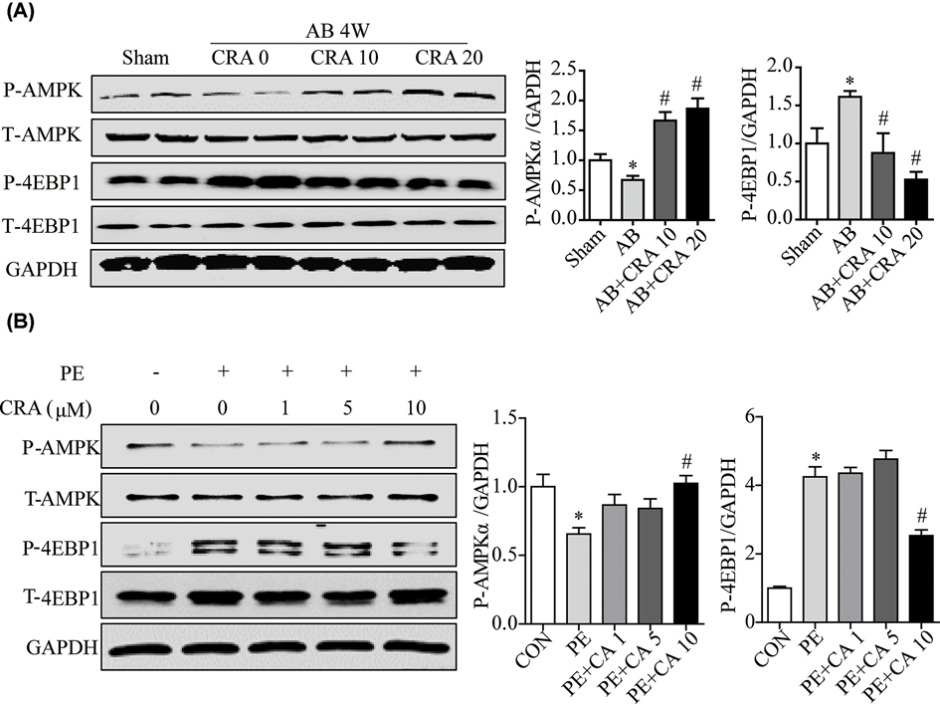
CRA induces P-AMPK activation and P-4EBP1 inactivation in cardiac hypertrophy (**A**) Representative blots and quantitative results for P-AMPK and P-4EBP1 protein expression in myocardium in each group (*n* = 6 per group). *P < 0.05 versus Sham group. #*P* < 0.05 versus AB group. (**B**) Representative blots and quantitative results for P-AMPK and P-4EBP1 protein expression in H9c2 cells in each group (*n* = 4 per group). *P < 0.05 versus CON group. #*P* < 0.05 versus PE group.

**Figure 8:**
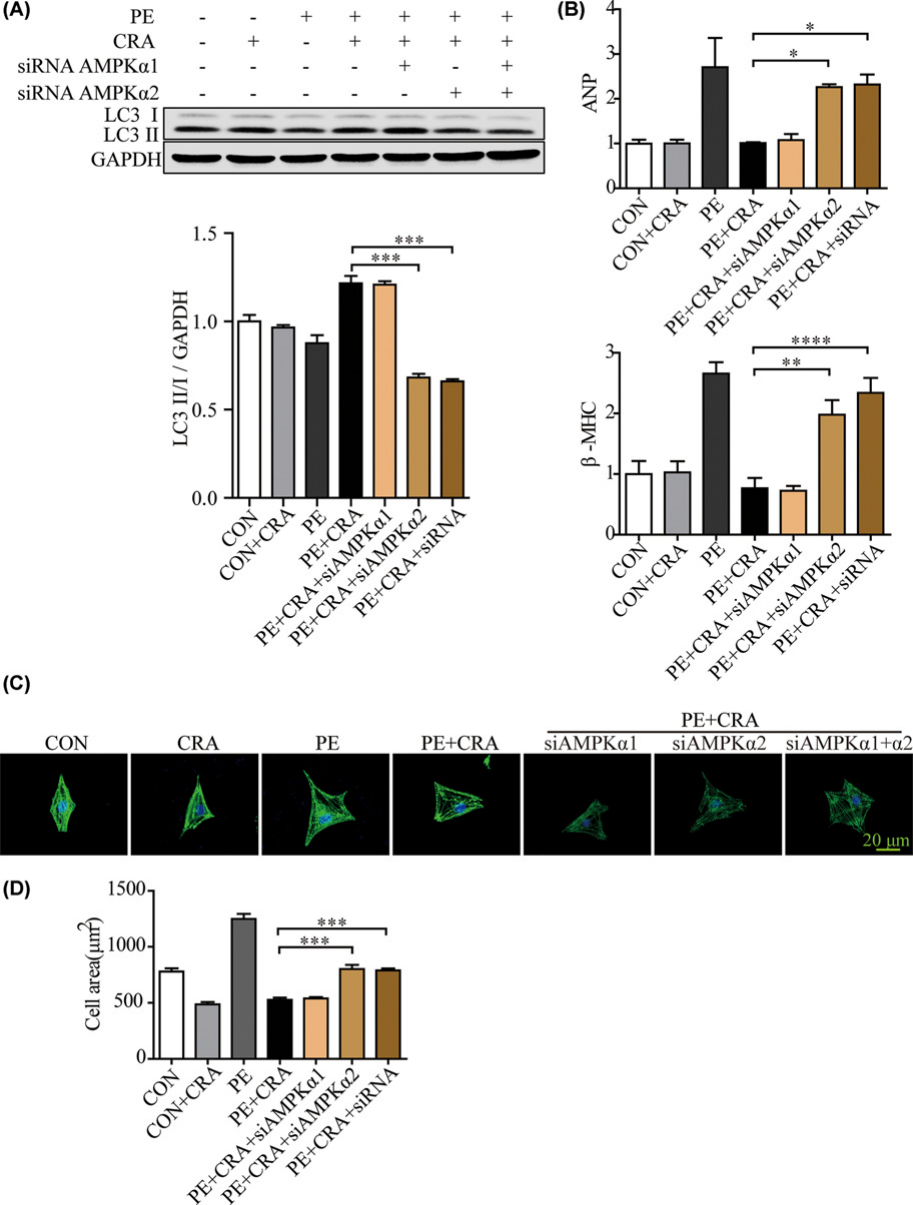
AMPK knockdown with AMPKα2 siRNA counteracted LC3 II expression increase and the hypertrophic response inhibition caused by CRA in PE-treated H9c2 cells (**A**) Representative blots and quantitative results for LC3 protein expression in H9c2 cells in each group (*n* = 4 per group). (**B**) ANP and β-MHC mRNA expression in H9c2 cells in each group. (**C**) Representative imaging of immunostaining H9c2 cells for α-actinin (green) in each group. (**D**) Quantification of cell surface area (*n* = 50^+^ cells per group); *P < 0.05, **P < 0.005, ***P < 0.0005, ****P < 0.0001.

We aim to illustrate the molecular mechanisms involved in the activation of autophagy by CRA in cardiac hypertrophy. Since CRA was found to activated AMPK in several tissues [7,14], and AMPK was a positive regulator of autophagy, we analyzed the protein expression of p-AMPK and p-4EBP1 in mice hearts and H9c2 cells in the indicated groups. We found that pressure overload-induced p-AMPK expression decrease and p-4EBP1 expression increase were significantly counteracted by CRA in heart tissue (Figure 7A). Consistently, PE-induced p-AMPK expression decrease and p-4EBP1 expression increase were significantly counteracted by CRA in H9c2 cells (Figure 7B). To confirm the roles of AMPK in CRA-treated H9c2 cells, we performed loss-of-function studies using AMPK siRNAs. AMPK knockdown with AMPKα2 siRNA counteracted LC3 II expression increase caused by CRA in PE-treated H9c2 cells (Figure 8A); moreover, the hypertrophic response to PE stimulation in PE+CRA+siAMPKα2 group but not PE+CRA+siAMPKα1 group was accelerated as compared with the PE+CRA group, as indicated by analyses of hypertrophic markers and cell surface area (Figure 8A–D). These results indicate CRA inhibits cardiac hypertrophy through regulating AMPK signaling pathways and autophagy.

## Discussion

At 4 weeks after AB, CRA-treated mice developed blunted cardiac hypertrophy, fibrosis, and dysfunction, and showed increased LC3 II and p-AMPK expression. It was also found that CRA blunted cardiomyocyte hypertrophy and promoted autophagy in Angiotensin II (Ang II)-treated H9c2 cells. In line with the *in vivo* studies, CRA also inhibited the hypertrophic response induced by PE stimulation accompanying with increased LC3 II and p-AMPK expression. It was also found that autophagy inhibition achieved by CQ (an inhibitor of the fusion between autophagosomes and lysosomes) or 3-MA (an inhibitor of autophagosome formation) counteracts the protective effect of CRA on cardiomyocyte hypertrophy. Interestingly, AMPK knockdown with AMPKα2 siRNA counteracted LC3 II expression increase and the hypertrophic response inhibition caused by CRA in PE-treated H9c2 cells.

Removal of harmful proteins and damaged organelles caused by autophagy could bring cell protection. But excessive autophagy beyond a certain level leads to deletion of necessary proteins or organelles, and results in cell death [3]. Autophagy exists constitutively in the normal myocardium and is substantially up-regulated and plays a protective role during the transition from hypertrophy to heart failure. After chronic pressure overload, the cardiomyocytes may suffer from autophagy flux insufficiency and the accumulation of autophagic vacuoles, and then cardiomyocyte hypertrophy occurs [15]. Four weeks of AB treatment induced significant LC3 II accumulation in the mouse myocardium, inhibition of AB-induced autophagy in WT mice by intraperitoneal injection of CQ led to deteriotation in cardiac hypertrophy and dysfunction [15]. CQ was an inhibitor of the fusion between autophagosomes and lysosomes, and 3-MA was an inhibitor of the autophagosomes formation. In our study, compared with the PE+CRA group, CQ or 3-MA inhibited the autophagy flux and increase the hypertrophic responses. The present study revealed that CRA improved autophagy flux, which was inversed in CQ or 3-MA-mediated LC3 turnover assays. AMPK is a critical sensor of the cellular energy status, activation of which has been proved to promote autophagy by inactivating mTORC1/4EBP1 [16] or directly phosphorylating a protein kinase, ULK1 [17]. Our findings suggest that CRA activated the AMPK pathway in hypertrophic cardiomyocytes. We also found that AMPK knockdown with AMPKα2 siRNA counteracted LC3 II expression increase and the hypertrophic response inhibition caused by CRA in PE-treated H9c2 cells. Therefore, the protective effect of CRA against cardiac hypertrophy is very likely due to AMPK-dependent autophagy.

A large body of studies investigated the role of autophagy in Ang II-induced cardiac hypertrophy and, but the conclusions from different reports are different. Some researchers thought that whether activation or inhibition of autophagy plays a protective role would depend on the model and the signaling pathway involved [18]. Some reported that the mildly enhanced autophagy in cardiomyocytes subjected to Ang II treatment was a compensatory protective mechanism, and further enhancing the compensatory autophagy of cardiomyocytes was protective [19]. More recent reports have overwhelmingly supported that myocardial ALP insufficiency results from chronic pressure overload and contributes to maladaptive cardiac remodeling and heart failure [4,20]. Several studies indicated that increased LC3II expression exists in cardiomyocytes under Ang II stimulation [21], in this condition, reducing LC3II, which presenting reducing the autophagosomes, may play protective role in Ang II-induced injury. We speculated that when there is an ALP insufficiency, reducing the numbers of autophagosomes may be beneficial. Consistent with the previous studies, in our study, Ang II increased autophagy in cardiomyocytes, what’s more, CRA treatment exaggerated the LC3II/I expression in cardiomyocytes after Ang II stimulation. Therefore, different stimulators lead to different changes in autophagy, and CRA bring beneficial effects on cardiomyocyte after PE or Ang II treatment.

In summary, our study revealed that CRA antagonized pressure overload-induced cardiac hypertrophy and PE-induced cardiomyocyte hypertrophy by positive regulation of autophagy. CRA may attenuate cardiac hypertrophy by regulating AMPK-dependent autophagy.

## Supplementary Material

Supplementary Figure S1

## Abbreviations

Ang II: Angiotensin II
CRA: corosolic acid
EF: ejection fraction
ESP: end-systolic pressure
EVD: end-diastolic volume
FS: fractional shortening
