# Supplementary material for: Corosolic acid ameliorates cardiac hypertrophy via regulating autophagy
Source: Biosci Rep. 2019 Dec 4;39(12):BSR20191860. doi: 10.1042/BSR20191860 (PMC6893168; doi:10.1042/BSR20191860)
Supplement: Supplementary Figure S1 [file BSR-2019-1860_supp.pdf]

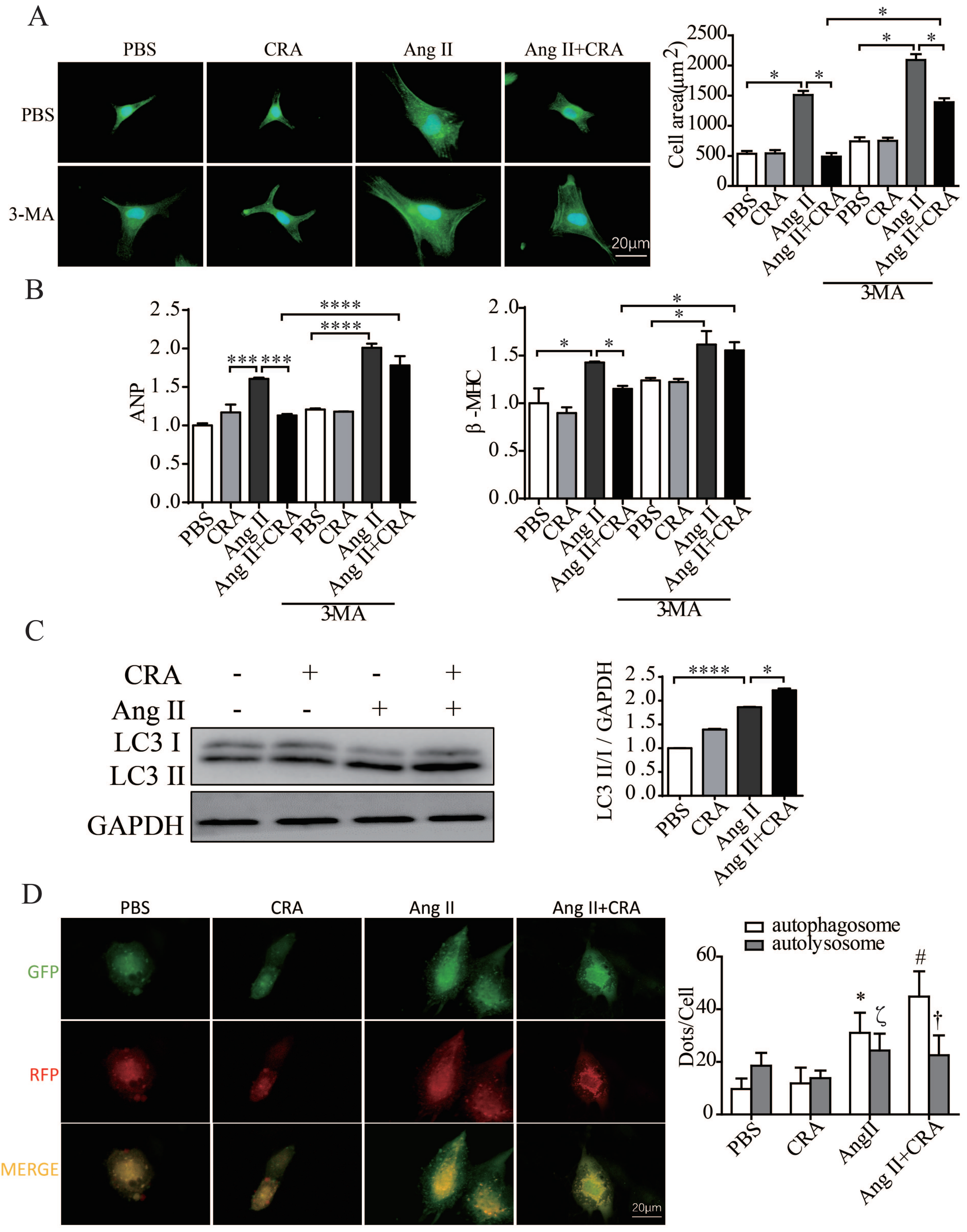
A
PBS
CRA
Ang II
Ang II+CRA
PBS
3-MA
20μm
Cell area(μm 2)
2500
2000
1500
1000
500
0
*
*
*
*
*
*
PBS
CRA
Ang II
Ang II+CRA
PBS
CRA
Ang II
Ang II+CRA
3MA
B
ANP
2.5
2.0
1.5
1.0
0.5
0.0
****
****
***
***
PBS
CRA
Ang II
Ang II+CRA
PBS
CRA
Ang II
Ang II+CRA
3MA
β -MHC
2.0
1.5
1.0
0.5
0.0
*
*
*
*
PBS
CRA
Ang II
Ang II+CRA
PBS
CRA
Ang II
Ang II+CRA
3MA
C
CRA - + - +
Ang II - - + +
LC3 I
LC3 II
GAPDH
LC3 II/I / GAPDH
2.5
2.0
1.5
1.0
0.5
0.0
****
*
PBS
CRA
Ang II
Ang II+CRA
D
PBS
CRA
Ang II
Ang II+CRA
GFP
RFP
MERGE
20μm
autophagosome
autolysosome
Dots/Cell
60
40
20
0
#
*
ζ
†
PBS
CRA
AngII
Ang II+CRA

Figure Legends

Figure S1. CRA blunts cardiomyocyte hypertrophy and promotes autophagy in Ang II-treated H9c2 cells. (A) Representative imaging of immunostaining H9c2 cells for α-actinin (green) and quantification of cell surface area (n=50+ cells per group)in each group. *P<0.05, ***P<0.0005, ****P<0.0001. (B) ANP and β-MHC mRNA levels in H9c2 cells in each group (n=6 per group). *P<0.05, ***P<0.0005, ****P<0.0001. (C) Representative blots and quantitative results for LC3 in H9c2 cells in each group (n=4 per group). *P<0.05, ***P<0.0005, ****P<0.0001. (D) Autophagy was evaluated by transfection with an adenovirus carrying mCherry-GFP-LC3 in each group (n=30 per group). *P<0.05 versus PBS group. ζ P<0.05 versus PBS group. # P<0.05 versus Ang II group. †P<0.05 versus Ang II group.
